# Weak Influence of Paleoenvironmental Conditions on the Subsurface Biosphere of Lake Ohrid over the Last 515 ka

**DOI:** 10.3390/microorganisms8111736

**Published:** 2020-11-05

**Authors:** Camille Thomas, Alexander Francke, Hendrik Vogel, Bernd Wagner, Daniel Ariztegui

**Affiliations:** 1 Department of Earth Sciences, University of Geneva, 1205 Geneva, Switzerland; daniel.ariztegui@unige.ch; 2 Department of Earth Sciences, University of Adelaide, 5005 Adelaide, Australia; alexander.francke@adelaide.edu.au; 3 Institute of Geological Sciences & Oeschger Centre for Climate Change Research, University of Bern, 3012 Bern, Switzerland; hendrik.vogel@geo.unibe.ch; 4 Institute of Geology and Mineralogy, University of Cologne, 50674 Cologne, Germany; wagnerb@uni-koeln.de

**Keywords:** bacteria, Archaea, glacial stages, lake sediment, deep biosphere

## Abstract

Lacustrine sediments are widely used to investigate the impact of climatic change on biogeochemical cycling. In these sediments, subsurface microbial communities are major actors of this cycling but can also affect the sedimentary record and overprint the original paleoenvironmental signal. We therefore investigated the subsurface microbial communities of the oldest lake in Europe, Lake Ohrid (North Macedonia, Albania), to assess the potential connection between microbial diversity and past environmental change using 16S rRNA gene sequences. Along the upper ca. 200 m of the DEEP site sediment record spanning ca. 515 thousand years (ka), our results show that Atribacteria, Bathyarchaeia and Gammaproteobacteria structured the community independently from each other. Except for the latter, these taxa are common in deep lacustrine and marine sediments due to their metabolic versatility adapted to low energy environments. Gammaproteobacteria were often co-occurring with cyanobacterial sequences or soil-related OTUs suggesting preservation of ancient DNA from the water column or catchment back to at least 340 ka, particularly in dry glacial intervals. We found significant environmental parameters influencing the overall microbial community distribution, but no strong relationship with given phylotypes and paleoclimatic signals or sediment age. Our results support a weak recording of early diagenetic processes and their actors by bulk prokaryotic sedimentary DNA in Lake Ohrid, replaced by specialized low-energy clades of the deep biosphere and a marked imprint of erosional processes on the subsurface DNA pool of Lake Ohrid.

## 1. Introduction

With an age of at least 1.36 million years (Ma) [1], Lake Ohrid (North Macedonia, Albania) is considered to be the oldest lake in Europe [1]. Owing to its age, location in the climate sensitive Mediterranean region, and its high degree of endemic biodiversity, Lake Ohrid has been targeted for a scientific deep drilling campaign co-sponsored by the International Continental Scientific Drilling Program (ICDP) in 2013. Global and regional scale changes in Pleistocene glacial–interglacial climatic boundary conditions exerted pronounced impacts on the terrestrial and aquatic environments in the lake and its catchment [1,2,3,4,5,6,7,8,9,10,11,12,13]. The main findings suggest that although significant environmental changes are recorded in the catchment and the sediments [2,4], no significant difference can be observed in lake organisms diversification rates [14,15], which suggests a high resilience of the Lake Ohrid’s ecosystem. In particular, diatom communities have been relatively stable for more than 1 Ma [16] and were shown to quickly return to a pre-disturbance state after significant tephra fallout from volcanic eruptions (Campi Flegrei caldera) without experiencing evident changes related to short-term climatic events (e.g., Heinrich H4 event) [15]. Similarly, diversification rates of endemic microgastropods were quite constant and led Föller et al. (2015) suggested that the specific bathymetry, tectonic activity and karst hydrology of Lake Ohrid could buffer environmental changes and contribute to the strong resilience of this ecosystem [17].

Among the organisms susceptible to environmental change in lake systems, prokaryotes have been the subject of increased attention in the past decade. Bacteria and archaea are present everywhere and are relatively sensitive to changes in organic matter inputs, lake stratification, temperature, pH and salinity of lake systems [17,18,19,20]. Consequently, the study of their diversity in lake sediments has become a means to understand their long-term response to environmental variations. In various lake systems, the living deep biosphere was shown to retain information on past climatic conditions [21,22]. In particular, deep scientific drillings into lake sediments have advanced our understanding of low energy systems and highly resilient subsurface microbial communities [23,24]. In Laguna Potrok Aike (Patagonia, Argentina) for example, microbial communities and their imprint differed from glacial to interglacial stages [25,26]. In Lake Van (Van, Turkey), changes in sulfate reduction rates were very sensitive to organic matter quality, varying as a function of changes in environmental conditions [27]. In the hypersaline conditions of the Dead Sea, strong similarities were observed between communities in sediments deposited in very arid conditions, while sediments deposited during more humid periods displayed apparent variability and diversified metabolic potential [28,29]. Such results, all originating from deep scientific drilling projects, have shown that the deep biosphere is a precious tool to evaluate and understand changes in paleoclimatic conditions, and permits estimates of the variable roles of microbes onto diagenetic processes [30,31] through time.

However, these studies are still scarce, and more analyses from other lakes are required to validate and potentially generalize the hypothesis of retained sensitivity of the lake subsurface biosphere to paleoclimatic conditions. Indeed, models and studies from other lakes, generally in shallower sediments, have emphasized the strong dominance of taxa adapted to low energy environments, similar to those found in ocean sediments [32]. A second hypothesis is therefore that eventually conditions become too exclusive (i.e., poor in nutrients and in labile organic matter) and result in the takeover of energy conservative slow-growing organisms such as Bathyarchaeota, Atribacteria, Dehalococcoidia or other poorly understood microorganisms likely better adapted to the specificity of deep sedimentary environments [32,33].

In order to test these hypotheses, we have explored the composition of 16S RNA gene sequences from prokaryotic DNA in several sediment intervals along the DEEP site drill core from the central part of Lake Ohrid. By comparing sedimentary microbial diversity and predicted functions with environmental parameters associated with this sediment, we attempted to find links and potential causality between the current structure of the deep biosphere, and the chemical and lithological characteristics of the sediment. We also compared this microbial composition with the magnetic properties of the sediment, as previous work has emphasized a strong shift in diagenetic paramagnetic minerals, likely caused by a change in microbial cycling in the subsurface sediments of the lake [11]. Finally, we investigated the relationship between prokaryotic diversity and paleoclimactic conditions using climate simulation and proxy observation data available for the past 1.36 Ma [1]. Studying the composition and current functions of the deep biosphere of Lake Ohrid should allow us to decipher whether microbes are more sensitive than eukaryotes to Quaternary changes in paleoenvironmental conditions, or if the low energy environments of the deep subsurface along with the lake system ability to buffer environmental changes has had a stronger impact and is selected for adapted taxa, regardless of the original depositional conditions.

## 2. Geological and Limnological Settings

**Figure 1:**
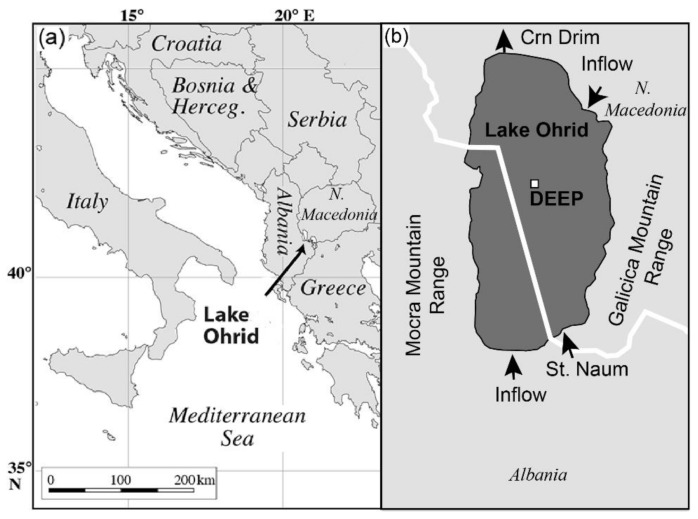
Map of the location of Lake Ohrid (**a**), and of the DEEP drilling site (**b**) at the border between N Macedonia and Albania.

Lake Ohrid covers an area of 358 km^2^ at the border between Albania and North Macedonia (Figure 1a). It is located in an N-S extending pull-apart basin, between the Galicica (East) and Mocra (West) mountain ranges (Figure 1b) at an altitude of 693 m above sea level (asl). Its mean water depth equals 150 m; the maximum reaches 293 m [34]. The lake is fed by karstic inflow (55%, [35]), partly originating from neighboring Lake Prespa located 10 km east of Lake Ohrid, small rivers, and direct precipitation on the lake surface. The high amount of nutrient-poor karst inflow results in an overall oligotrophic status of the lake. 

The DEEP drilling site is located at 243 m water depth, in the central part of the lake (41°02′57″ N, 020°42′54″ E, Figure 1b). During the drilling in 2013, several cores were recovered at this site, reaching a terminal depth of 569 m below lake floor (mblf, [36]). The upper 200 m of the DEEP site composite core analyzed herein is composed of a succession of fine grained hemipelagic sediments, with a few (generally less than 5 cm thick) intercalated event layers classified as mass wasting deposits and tephra in the presence/absence of microscopic glass shards [2,37]. Three lithotypes were identified in the fine-grained sediments, based on the amount of calcium carbonate: calcareous silty clay, slightly calcareous silty clay and silty clay. These variations are reflected in the calcite and total organic carbon (TOC) content of the deposits. Silty clayey sediments are mostly characterized by low organic matter (OM) concentrations, while OM can be moderate to high in calcareous and slightly calcareous sediments. The sediments appear mottled or massive and lamination is absent, which implies bioturbation and oxygenated bottom water conditions at the time of deposition [2].

In silty clay and slightly calcareous silty clay, TOC is predominantly of aquatic origin, as inferred by the carbon-to-nitrogen ratio (C/N) [38], while sediments from calcareous silty clay show C/N ratios occasionally above 10, implying somewhat elevated terrestrial OM inputs. Overall, the C/N ratio and δ^13^C of TOC and TIC at the same site support a dominance of OM from aquatic primary production [8]. However, Francke et al. (2016) suggest that these values may be affected by early diagenetic selective N loss, since the DEEP site is almost completely disconnected from the inlet stream supply. Rock-Eval analyses on a Late Glacial to Holocene sediment succession retrieved close to the Lini Peninsula (2.5 km to the west of the DEEP site) revealed organic matter mainly of aquatic origin [3]. Lipid biomarker analyses on sediments of a similar age retrieved in close proximity of inlet streams however yielded dominance of terrestrial organic endmembers [39], which is also supported by C/N ratios >10 in surface sediments close to the major inlets [40].

High diatom frustules content, high endogenic calcite concentrations, and overall high OM in the core corresponds to periods of higher primary productivity, likely promoted by higher temperatures and increased supply of nutrients and dissolved ions (Ca, CO_3_) from the (karst) catchment, i.e., conditions as they mainly occur during wet and warm interglacial periods. On the opposite, lower OM, endogenic calcite, and biogenic silica contents were interpreted as periods of lower productivity, coupled with increased OM oxidation and mixing during the winter season [2,5,8]. These conditions are primarily characteristic of glacial dry and cold periods [2,5].

## 3. Materials and Methods

### 3.1. Sampling Material

Samples for microbial and sediment biogeochemistry analysis were taken from core catchers originating from hole 5041-1B. Immediately after core retrieval, mini cores were taken from the core catchers using pre-cut and autoclaved (sterile) syringes for microbial analyses. This was done for every available core-catcher (at ca. 3 m resolution, representing the length of one most core runs) in the DEEP core, by carefully avoiding contamination from core liners and drilling apparatus [23]. These minicores were then stored at −12 °C until further processing. The ages of the core catcher sediment samples of core 5045-1B were inferred from the published age model [1]. 

### 3.2. Sediment Chemistry

Biogeochemical data of core catcher samples presented herein were previously published [34]. After freeze-drying, total carbon (TC) and total inorganic carbon (TIC) were analyzed as released CO_2_ from powdered material using an DIMATOC 200 (DIMATEC, Essen, Germany.) TOC was calculated as the difference between TC and TIC. Total nitrogen (TN) concentrations were analyzed using a Vario MicroCube. 

X-ray fluorescence (XRF) analyses were carried on freeze-dried, powdered aliquots (1 g) of the core catcher samples using an ITRAX core scanner (Cox Analytical Systems, Mölndal, Sweden). The ITRAX core scanner was equipped with a chromium (Cr) X-ray source and was run at 30 kV and 30 mA, with an integration time of 10 s. Data processing was performed with the QSpec v. 6.5 software (Cox Analytical).

Magnetic property data were taken from Just et al. (2016). Climatic data (including simulated precipitation and temperatures) were taken from Wagner et al. (2019). 

### 3.3. DNA Extraction and Sequencing

Half a cubic centimeter of wet sediment was extracted for 36 samples, using the MOBIO powersoil extraction kit (Qiagen, Valencia, CA, USA). We realized triplicate DNA amplification of ca. 10 ng of DNA per triplicate using universal primer 515F (5′-GTGYCAGCMGCCGCGGTA-3′) and 909R (5′-CCCCGYCAATTCMTTTRAGT-3′) for the V4–V5 hypervariable region of the 16S rRNA gene [41], with indexes integrated following the dual-indexing procedure described by [42]. Pooled triplicate products were then quantified using Picogreen assay (Life Technologies, Carlsbad, CA, USA)) and pooled equimolarly (same amount for each sample). The final pool was concentrated with SpeedVac Plus SC110A Savant and purified with CleanNA beads (CleanNA, Waddinxveen, the Netherlands) before sequencing was performed by Fasteris (Geneva, Switzerland) on an Illumina Miseq with 2 × 250 cycles, with settings of 7.5 Gb yield (including PhiX), an error rate of 2.5% (within quality specifications) and Q30 at 75% (Illumina, San Diego, CA, USA). 

### 3.4. DNA Sequence Processing

The workflow included adapters removal using trimmomatic (v. 0.32) [43], paired-ends reads joined with ea-utils (v. 1.1.2-537) [44], quality-check using FastQC (v. 0.11.5), and sample demultiplexing by Fasteris in-house script. 16S rRNA gene sequences were then processed using Mothur v.1.41.1 [45]. Samples were dereplicated, aligned, and filtered by length. Chimeras were removed using uchime (v. 4.2.40) [46], and taxonomic affiliation was then performed using the method of [47] at a cutoff of 80% against the Silva SSU database 132 [48]. Known common contaminants were removed by hand using the list provided by [49], and a custom list of sequences obtained from extraction blanks. Operational taxonomic units (OTU) were then defined at 80%, 95% and 97% similarity cutoff and used for similarity analysis (details in next section). Random subsampling was realized based on the smallest number of obtained sequences in one sample after singleton removal for diversity analyses at the 97% OTU level.

All alpha-diversity indexes were calculated based on 97% OTU matrix using Mothur. The local and species contribution to beta-diversity (LCBD and SCBD respectively) were calculated from the same matrix with R (v. 3.6.3) using formula provided by [50].

### 3.5. Data Analysis

Except for the 16S rRNA barplot, which was built using decontaminated 16S rRNA gene sequence taxonomy reads at the phylum level, multivariate analyses presented in this article were realized using OTU matrices (sample vs. OTU). Three OTU definitions were used at cutoffs of 80%, 95% and 97%, based on the similarity cutoff for phylum, class and species level, respectively [51]. 

For each cutoff, dissimilarity matrices (non-metric multidimensional scaling) and hierarchical cluster analysis were built from a standardized OTU matrix using Bray Curtis similarity, 999 permutations and 5% significance level using the Primer7 software. Similarity percentages (SIMPER) and associated OTU that had a high contribution in defining these groups were compiled using Bray–Curtis similarity with a cut-off at 70% for low contributions, and the contribution of the aforementioned significant OTUs were presented in a heatmap along with the hierarchical cluster for samples.

Environmental variables were pooled into three different matrices (lithology, magnetic properties and simulated climatic variables, the latter encompassing modeled air temperatures and precipitations), normalization and a principal component analysis were obtained using the software PAST (v. 4.03) [52]. The matrices were then fitted to the Hellinger transformed OTU dissimilarity matrix with 9999 permutations. We constrained the dissimilatory matrix by running a distance-based redundancy analysis (db-RDA) using Bray–Curtis distance and assessed the significance of the variables using ANOVA tests with 5000 permutations. We then used a forward selection to confirm significant variables and plotted them using obtained axes values.

Finally, potential functions were obtained using the online tool METAGENassist [53] based on taxonomic affiliation of obtained OTUs. A heatmap was built using Pearson distance and Ward clustering algorithm after unmapped and unassigned reads were excluded, along with OTUs appearing in only 10% of the samples. Data filtering was done using interquantile range. Row-wise (sample by sample) normalization was performed using the median, while column-wise normalization was done by auto-scaling (mean-centered and divided by the standard deviation for each variable).

The complete list of OTUs and sequences can be downloaded from NCBI Genbank (MT066494–MT067558). Environmental parameter matrices are available as supplementary material (Tables S1–S5) and R and Mothur scripts are available on the Open Science Framework data repository public webpage (https://osf.io/s9e2q/). 

## 4. Results

### 4.1. Lake and Sediment Characteristics

**Figure 2:**
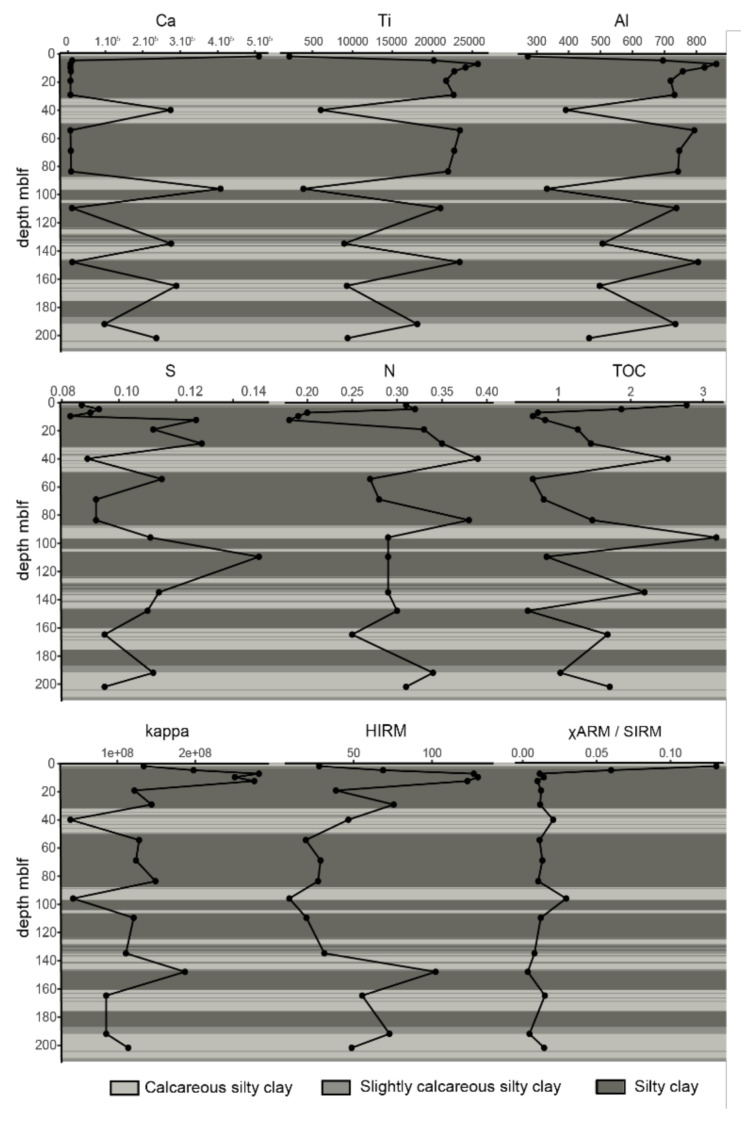
Profiles of elemental composition and ratio along the core, with corresponding sedimentary facies as described by Francke et al. (2016).

**Figure 3:**
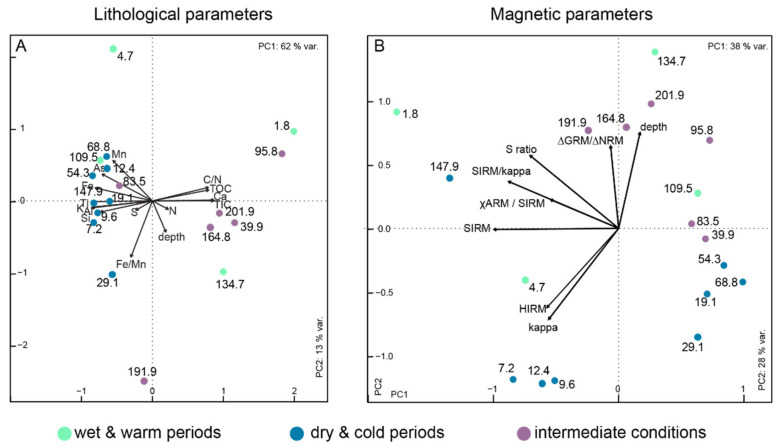
Principal component analysis of elemental composition of the core (**A**) and magnetic properties (**B**) along the core. Numbers correspond to sample depth (in m), and color code for wet and warm periods, mainly corresponding to interglacials (green), dry and cold periods generally corresponding to glacial stages (blue), and intermediate conditions for transitional climatic stages (purple), based on data by [1,2,8,11]. HIRM hard isothermal remanent magnetization; SIRM soft isothermal remanent magnetism; ARM anhysteretic remanent magnetization; NRM natural remanent magnetization; GRM gyro remanent magnetization; kappa magnetic susceptibility; S ratio: proportion of high to low-coercivity magnetic minerals, see Just et al., 2016 for more details.

Due to the low sampling resolution, sediment geochemical characteristics display a relatively scattered pattern along depth (Figure 2) but conserve a strong relationship with climatic patterns (strong clustering of dry-cold periods) (Figure 3). A plot of the principal components explaining 62 and 13% of the variance shows that TIC and Ca vary together (Figure 3). TOC and the C/N ratio also have a similar behavior. Detrital elements Ti, K, Al and Si are anticorrelated to TOC, Ca and TIC. Fe, As and Mn have quite similar behavior with each other, but seem not to be correlated to sediment depth. Overall, there is a marked distinction between samples that have high TOC, C/N ratio, Ca and TIC, and others that have higher Mn, Fe, Ti, K, Al and Si values. The former mainly belong to interglacial stages, while the second are generally from glacial periods. The N profile is relatively similar to TOC in the first 80 m but differs below. The S profile also differs from all the others with variations unrelated to facies composition (Figure 2). Three remarkable samples can be identified based on their environmental parameter characteristics: the samples at 191.9 m and 29.1 m, which have high Fe/Mn ratio values, and the sample at 4.7 m, which has low Fe/Mn. 

Magnetic properties have been described in detail in Just et al. (2016). The displayed principal components here account for 28% and 38% of the variance of selected magnetic properties (Figure 3). The PCA reveals here that magnetic susceptibility (kappa) and hard isothermal remanent magnetization (HIRM), which integrates the participation of high-coercivity magnetic minerals (like hematite or goethite) to the saturation isothermal remanent magnetization (SIRM), behave similarly. They all show how detrital magnetic minerals have accumulated preferentially in the sediments during dry and cold periods. They show a slight anticorrelation with depth. The other properties seem independent from each other, and no clear clustering can be observed for the samples. Samples between 4.7 m and 29.1 m are characterized by high kappa and HIRM. Samples below 95.8 m bear a higher imprint of greigite, marked by high ∆GRM/∆NRM. No clear distinction is observed in terms of glacial vs. interglacial stages. Finally, χARM/SIRM, which can be used to estimate magnetic grain size, and has been shown to potentially illustrate the presence of microbial greigite or magnetite [11], shows a maximum at the surface (0.13) and stable low values (ca. <0.02) below 5 m (Figure 2). 

### 4.2. Microbial Community Composition and Variation

**Figure 4:**
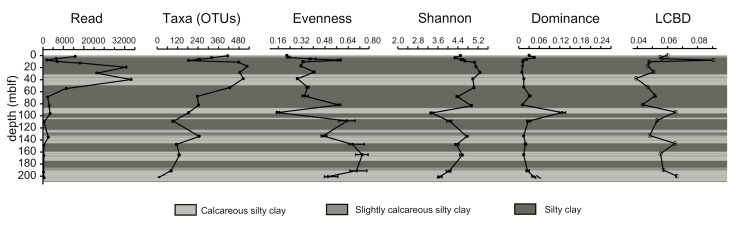
Diversity profiles including sequencing read number, OTU number, OTU richness, Shannon diversity index, evenness and local contribution to beta-diversity (LCBD) along the core (for OTUs defined at 97%), with corresponding sedimentary facies as described by Francke et al. (2016).

Out of the 36 intervals for which DNA was extracted, 18 only yielded usable 16S rRNA gene amplicons. The number of reads obtained from the profile varies largely (from 38,092 at 1.8 to 238 at 191.9 m) and has to be taken into account when analyzing the structure of the community. Reads drop significantly with depth, in particular below 60 m (Figure 4). This distribution is correlated with the decrease in the number of taxa (OTUs), although it is not exactly similar. However, diversity indexes are not related to read numbers. Evenness steadily increases with depth, but the Shannon index remains close to 4.5 and even 5 throughout the core, except for 95.8 and 201.9 m, where it drops below 4. Local contribution to beta-diversity peaks at 9.6 m in association with an increase of evenness. It then sharply decreases and follows a general increasing trend with depth, with a second maximum at 95.8 m correlated to high dominance and minimum evenness.

**Figure 5:**
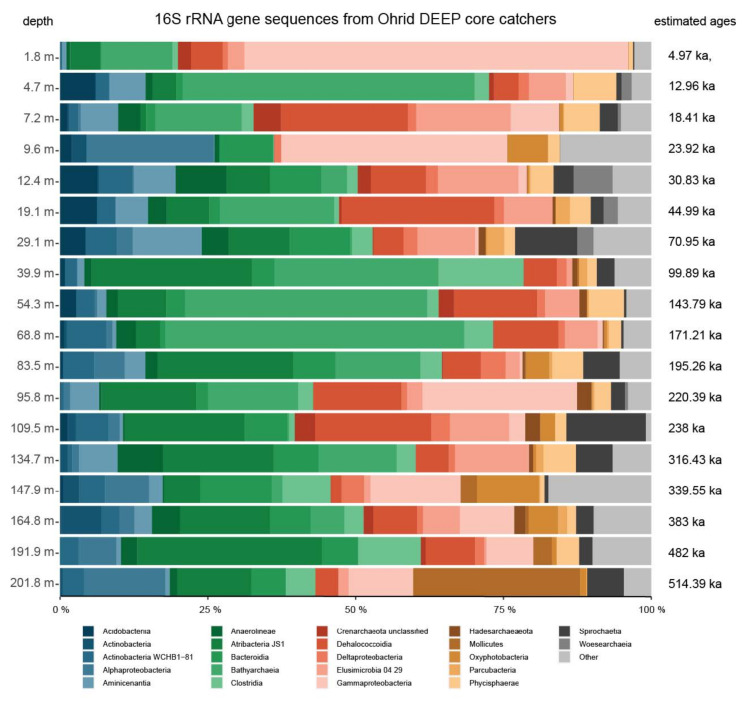
Relative abundance of 16S rRNA gene sequences per sample at the phylum level, and corresponding estimated ages for each sample.

**Figure 6:**
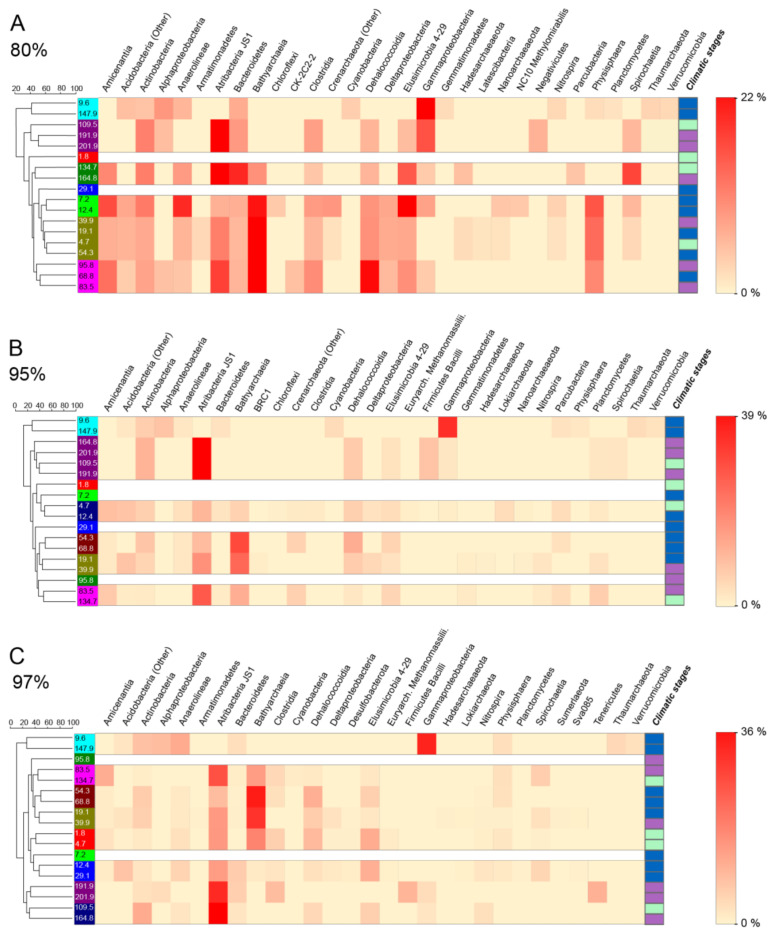
Heatmap of the relative contribution of significant OTUs (gathered under their taxonomic affiliations using SILVA) for each group defined by SIMPER, at (**A**) 80% cutoff for OTU definition, (**B**) 95% cutoff for OTU definition and (**C**) 97% cutoff for OTU definition. Sample groups share similar colors on the left side of the panel. Climatic stages are displayed for information using similar colors as in Figure 3.

**Figure 7:**
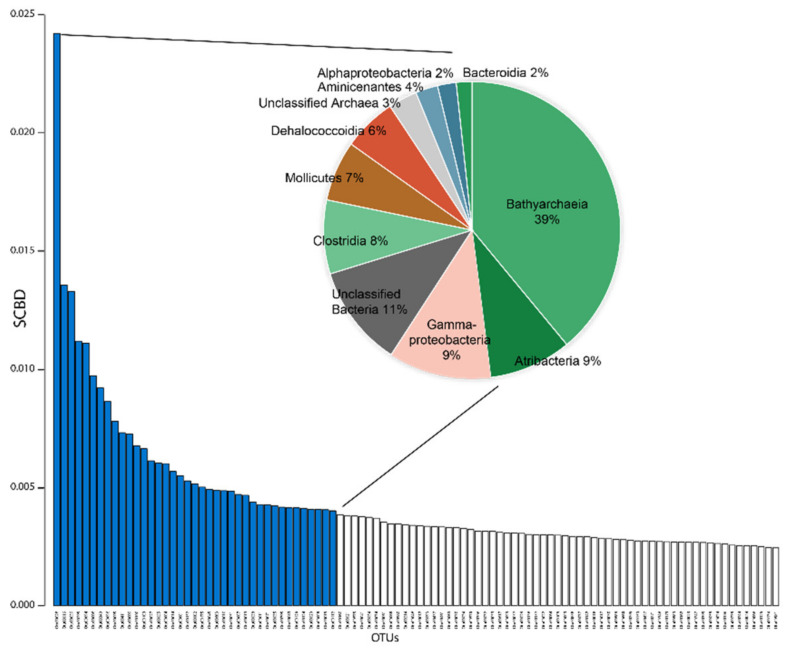
Species contribution to beta-diversity (SCBD) per OTU, and contribution and taxonomic assignment of the 40 first OTUs. Colors are the same as those used in Figure 5.

Based on phylum level barplots and three different OTU cutoffs, we identified three main taxa that significantly drive the structure of the deep biosphere community of Lake Ohrid: Atribacteria, Bathyarchaeia and Gammaproteobacteria (Figure 5 and Figure 6). Species contribution to beta-diversity is mostly carried by OTUs associated with Bathyarchaeia (39% of the first 40 OTUs contributions), with Atribacteria (9%), Gammaproteobacteria (9%) and Clostridia (8%) having an important contribution too (Figure 7).

Other phyla show significant relative percentages: Dehalococcoidia, Physisphaera, Elusimicrobia and members of Actinobacteria, and to a lesser extent Spirochaetia, Bacteroidia and Cyanobacteria (Oxyphotobacteria) (Figure 4). Three samples are characterized by a dominance of Gammaproteobacteria: 1.8 m, 9.6 m and 147.9 m (Figure 5 and Figure 6B,C). Atribacteria OTUs define samples below 83.5 m, while Bathyarchaeia OTUs seem more important above this depth. Other phyla like Dehalococcoidia, Elusimicrobia or Spirochaetia vary a lot with depth, SIMPER analyses at the three cutoff percentages have identified varying clusters of samples based on the OTU compositions (Figure 6). Samples at 9.6 m and 147.9 m always form a distinct group based on their high Gammaproteobacteria abundance and occurrence of Alphaproteobacteria, Actinobacteria, Anaerolineae, Thaumarchaeota, Cyanobacteria and Verrumicrobia-affiliated OTUs. A second group is formed by samples at 109.5 m, 191.9 m and 201.9 m (and 164.8 m for 95 and 97% OTUs) and characterized largely by Atribacteria JS1 related OTUs and to a lesser extent by Actinobacteria and Dehaloccocoidia. This group is actually divided into two subgroups at the 97% OTU cutoff based on a higher importance of Tenericutes, Firmicutes Bacilli and Clostridia at 191.9 m and 201.9 m (Figure 6C). For the other samples, groups based on the importance of OTUs vary from one OTU cutoff to another, and do not significantly represent given depth intervals, or similar types of climatic stages.

**Figure 8:**
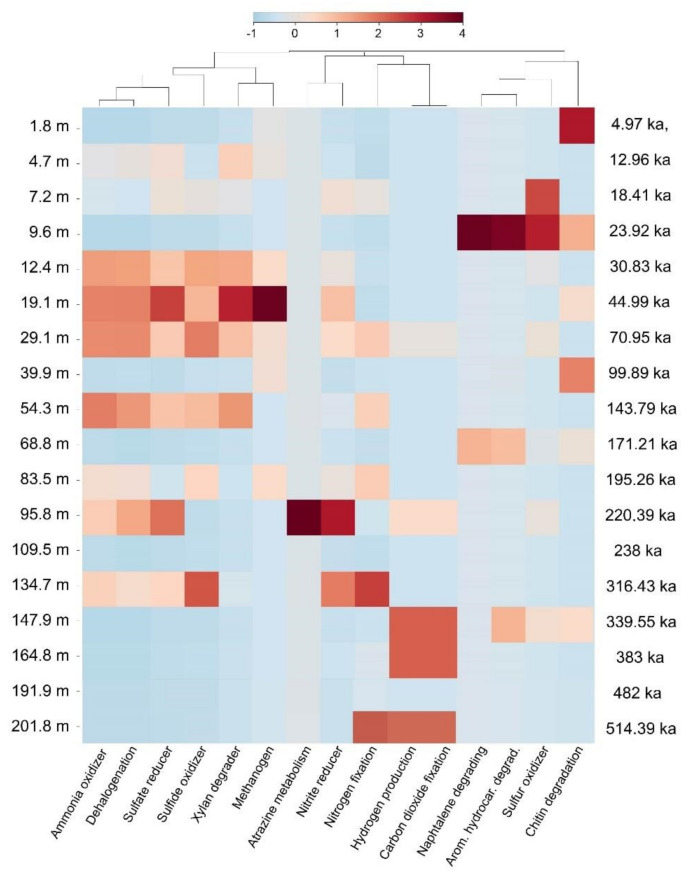
Heatmap of potential microbial metabolisms predicted for each sediment depth using the bioinformatics tool METAGENassist. Note that 25% of OTUs recovered were included in this analysis. Color scale of METAGENassist-provided normalized OTU abundances from −1 to 4.

Results from METAGENassist analyses only allowed assignment of metabolic functions to a rather small percentage of OTUs (25% for metabolisms), which prevents a complete and unbiased understanding of the metabolic capabilities of observed taxa. They show that samples at 7.2 m, 9.6 m and 12.4 m have a higher proportion of organisms associated with aquatic habitats. Higher sporulation is observed for deep samples at 109.5 m, 191.9 m and 201.9 m, along with enhanced motility (147.9 m, 191.9 m and 201.9 m). Just like diversity, large variations are observed for metabolisms (Figure 8) for OTUs that were attributed a function. Sulfate reducers and sulfide oxidizers dominate between 12.4 m and 29.1 m, and in samples at 54.3 m. Dehalogenation follows a similar occurrence. Sulfate reducers are also largely present at 95.8 m along with nitrite reducers. Sulfate reducers are dominant at 134.7 m, along with N fixers and nitrite reducers. CO_2_ fixation seems to dominate in the deep layers at 147.9 m, 164.8 m and 201.8 m. Hydrogen production is always co-occurring with this CO_2_ fixation. Finally, methanogens are observed between 12.4 m and 39.9 m.

**Figure 9:**
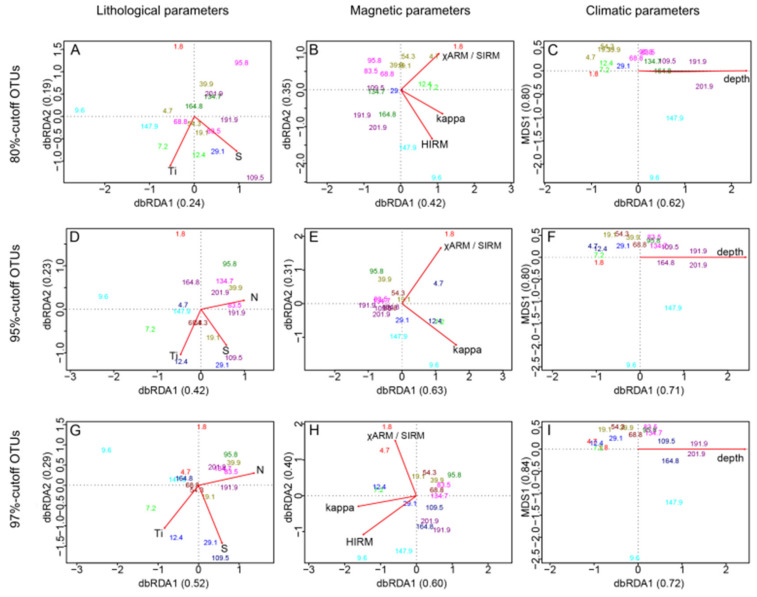
Correlation analysis of OTU distribution and environmental variables as defined using db-RDA and ANOVA test [1,2]. Panels (**A**–**C**): using 80% similarity OTU cutoff, (**D**–**F**): using a 95% similarity OTU cutoff, and panels (**G**–**I**) using a 97% similarity OTU cutoff. Left column shows lithological parameters, central column shows magnetic parameters and right color shows simulated climatic parameters. Only variables that showed significance through ANOVA tests are displayed. The color code is defined by cluster analysis and provided in Figure 6.

We plotted distance-based RDA analyses of OTUs with environmental parameters selected as significant by ANOVA tests (Figure 9). Regardless of cutoffs, the dbRDA analyses and ANOVA tests are relatively similar from one another. We found that for each OTU cutoff used, Ti and S were identified as significant parameters (Pr < 0.05) with N added as a third significant elemental parameter by forward selection for cutoffs at 95 and 97% (Figure 9C,G). Samples at 12.4 m, 29.1 m and 109.5 m are marked by high Ti and S. Nitrogen is particularly low for samples at 9.6 m and 7.2 m while clusters of samples at 191.9 m, 201.9 m, 83.5 m and 134.7 m are characterized by high N. For magnetic parameters, χARM/SIRM and magnetic susceptibility (Kappa) were found to be significant variables for all three cutoffs. HIRM was also added to significant parameters by forward selection for 80% and 97% OTU cutoffs (Figure 9B,H). Samples at 1.8 m and 4.7 m were particularly characterized by high χARM/SIRM, while samples at 12.4 m and 7.2 m had relatively high kappa, and 9.6 m and 147.9 m seem to carry high HIRM overall. Finally, the dbRDA analyses for climatic variables did not yield significant parameters other than depth (Pr = 0.001).

## 5. Discussion

### 5.1. Dominant Taxa and Associated Metabolisms in the Ohrid Sediment

Lake Ohrid’s sediments bear an original and diverse subsurface microbial community, based on the analysis of 16S rRNA gene sequences (Figure 4 and Figure 5). Three main phyla have been identified, two from the bacterial domain and one from the archaeal domain (Figure 5). Gammaproteobacteria seem to play a significant role in the structuration of the subsurface community and mainly occur in two specific samples that largely differ from the others (i.e., 9.6 m and 147.9 m; Figure 5 and Figure 6). These two samples have different taxonomic compositions resulting in different outcomes in terms of metabolic prediction (Figure 8). While the 9.6 m sample seems to be dominated by naphthalene, chitin and aromatic hydrocarbon degradation, the 147.9 m sample mainly exhibits hydrogen production and carbon dioxide fixation. The 9.6 m sample seems to be dominated by an oxic habitat community (as suggested by the varied organic matter degradation metabolic capacities outlined by METAGENassist, Figure 8). The contribution of soil-related OTUs (e.g., Acidobacteria, Actinobacteria or Bacteroidetes) also suggest that most of the DNA extracted from this sample associates with high fractions of terrestrial OM, thereby also containing soil microbes masking the subsurface biosphere contribution in this level. Conversely, this sample exhibits minimum TOC that could coincide with oxidative conditions at the time of deposition [2]. Hence, we suggest preservation of ex-situ microbial DNA rather than this sample being representative for an in situ sedimentary microbial community. 

The two other most significant phyla observed in Ohrid sediments belong to the archaeal candidate division Bathyarchaeia and the bacterial division Atribacteria. These are both common phyla in sedimentary environments at depth [54], and particularly in the marine realm (e.g., 32), where their occurrence has been associated with strong adaptations to low energy environments and varied fermentative abilities. Atribacteria have been suggested to perform primary fermentation of carbohydrates and secondary fermentation of organic acids (propionate among others), leading to the production of H_2_ [33,55]. Bathyarchaeia are more enigmatic as they have been hypothesized as organoheterotrophic and autotrophic acetogens [56], potentially able to perform dissimilatory nitrite reduction to ammonium. Lloyd et al. (2013) [57] also suggested they could degrade detrital proteins. Finally, CH_4_ production was also hypothesized for this clade [58]. These two phyla appear as the most important contributors to beta-diversity among the 40 first OTU contributions to SCBD (Figure 7). Their contribution as obtained from SIMPER analyses is also straightforward as they have the maximum participation in defining similar groups among all samples (Figure 6). They likely play a strong role in the deep subsurface of Lake Ohrid and are often associated with Dehalococcoidia phylum sequences, which form a common deep biosphere clade, in particular in marine sediments. Kawai et al. (2014) [59] hypothesized anaerobic respiration of organohalides for the Chloroflexi clade, but their catabolic reductive dehalogenation ability has been questioned by the study of several assembled genomes, which suggest they had a strictly anaerobic organotrophic or lithotrophic lifestyle. Sewell et al. (2017) [60] suggested their involvement in reductive dehalogenation with H_2_ as an electron donor and linked them to homoacetogenic Chloroflexi, which could connect their activity to other deep biosphere taxa like H_2_-producing Atribacteria, often presented as syntrophs [55]. Samples that have high Atribacteria and Bathyarchaeia relative abundance often bear reads associated with Deltaproteobacteria, Aminicenantes and Bacteroidetes (Figure 5 and Figure 6). Their metabolic abilities cannot be easily constrained using our method, but their occurrence has often been acknowledged in the deep subsurface [33]. Potential association with sugar fermentation coupled with Mn and Fe reduction was hypothesized for Bacteroidetes members [32] but is not expressed in the METAGENassist simulation (Figure 8). However, they likely have energy conservative metabolisms allowing them to remain present in extreme deep lacustrine sediments [26]. Based on sedimentary intracellular DNA analysis, Deltaproteobacteria, Bathyarchaeia and Clostridia were shown to be part of the increasing communities with depth in ferruginous Lake Towuti (Indonesia), suggesting they are well adapted to the deep subsurface environment [61]. 

Based on our METAGENassist simulation (Figure 8), samples between 12.4 m to 29.1 m and at 54.3 m carry a strong similarity in metabolic potential, encompassing ammonia oxidation, dehalogenation (likely supported by Dehalocccoidetes), sulfate reduction, sulfide oxidation, xylan degradation and methanogenesis. The unlikely co-occurrence of metabolisms predicted at this sediment depth suggests that archived DNA originating from the water column and catchment are over-represented in this sample. Apart from the fact that a major fraction of observed OTUs could not be linked to any functional potential, it is likely that our METAGENassist simulation is biased by the contribution of archived sedimented DNA from the catchment and water column. It could be the case for soil-derived Acidobacteria, or water-derived Alphaproteobacteria or Physisphaera for example. The contribution of Gammaproteobacteria and Cyanobacteria suggests likewise. 

### 5.2. Diversity Changes by Depth

Although depth is a significant parameter in structuring the community (Figure 9), the relative abundance of common deep subsurface taxa such as Bathyarchaeia or Dehalococcoidia does not exhibit a clear trend, except for Atribacteria (Figure 5), which tends to increase in relative abundance with depth (below 10 m). This is reflected in the varied alpha and beta-diversity indexes (Figure 4). Regardless of the number of OTUs, the Shannon index remains relatively high although a gentle decrease is observed with depth and corresponds with an increase of evenness that should be associated with the increasing contribution of energy-conservative taxa. A decrease in read number also suggests biomass and DNA quality decrease with depth. This is similar to the diversity profiles observed down to 80 m in freshwater lake Laguna Potrok Aike [26]. However, it is worth noticing that this diversity is lower compared to what has been observed in shallow lake sediments (first meter) [31,62]. Local contribution to beta-diversity is very high for the sample at 9.6 cm, as expected given its peculiarity in microbial community. Below 40 m, a general increase can be observed towards the deepest layers, which could be associated with a general depletion of less adapted taxa and a relative increase in the low-energy taxa such as Bathyarchaeia members, which carry much of the SCBD (Figure 7). Downcore, we conclude that we tend to lose the diversity that was originally provided by the sedimenting DNA in paleolake Ohrid. Energy conservative, well-adapted slow growers common in deep subsurface environments necessarily take over in terms of relative abundance, as described by Kirkpatrick et al. [63] in the marine realm, or Wurzbacher et al. [32] in lake deep sediments. Multivariate analyses coupled with ANOVA tests identified a series of external parameters that were significantly linked to given OTU relative abundances. In particular, Atribacteria were identified as being increasingly dominant with depth. Metatranscriptomics, metabolomics and single cell genomics studies from deep sediments of the Baltic Sea have highlighted the adaptations and metabolic activity allowing Atribacteria to remain active in low energy environments like the deep sediments of Lake Ohrid, e.g., the ability to produce de novo amino acids and export them in very low energy environments, likely halting cell growth and suggesting metabolic interdependencies [64].

The decrease in reads suggests lower quality of DNA with depth, which may result in an increased contribution of sedimented and extra-cellular DNA sources. Potentially, un-discarded (poorly-resolved) lab contaminants could also play a larger role in low-DNA samples like the deeper ones, and would shape the apparent diversity consequently. The increased contribution of Gammaproteobacteria might for example be partly associated with this pattern, but the poorly resolved taxonomic affiliations of most of their affiliated OTUs prevents further interpretation on this basis.

### 5.3. Impact of Environmental Parameters on Current Communities

Samples between 12.4 m and 29.1 m, and at 54.3 m, are all from cold and dry intervals. They exhibit a mix of metabolic potential involving anaerobic and aerobic processes (Figure 8). While anaerobic degradation processes coincide with sedimentary conditions, the presence of sequences associated with aquatic habitats, xylan degraders, nitrogen fixers, Gammaproteobacteria and Cyanobacteria (e.g., 9.6m and 147.9 m) fits quite well with the Ohrid depositional model in which glacials are characterized by lower productivity, well oxygenated water column and enhanced erosion and potential input of soil organic matter from the catchment [65]. This also coincides with low TOC, TIC and C/N levels, and high Ti, which have been associated with glacial stages with lower productivity and enhanced detrital inputs in Lake Ohrid [2,8]. The contribution of Ti and N is likely associated with such a pattern (Figure 9A,D,G). Consequently, the obtained DNA in these layers could result in a mix of archived sedimentary DNA, and active OM anaerobic degraders. 

Minerals carrying high HIRM are expected to occur in situations of enhanced erosion, either during glacial intervals or periods of low insolation during interglacials, when soil erosion was facilitated by lower vegetation cover [5,11,65]. High kappa, and HIRM is therefore likely related to increased erosion, and is reflected by the increased contribution of OTUs associated with transported and archived DNA (e.g., Gammaproteobacteria, Cyanobacteria, Acidobacteria and Actinobacteria; Figure 6 and Figure 9B,E,H). Of special interest is the occurrence of Cyanobacteria in samples at 9.6 m dated at 24 ka and at 147.9 m at 340 ka (Figure 6A,B). As Cyanobacteria are not expected to be active in the deep sediment, relative cyanobacterial increase in samples from glacial periods is likely associated with an increase in archived fossil DNA. In temperate lakes, limited nutrient and in particular N-deficiency has consensually been shown to support blooms of nitrogen-fixing Cyanobacteria [66,67]. This could explain the increased presence of Cyanobacteria in the 9.6 m and 147.9 m samples of Lake Ohrid, along with low C/N ratio [68], since dry and cold conditions during glacial periods likely caused nutrient depletion in the lake [7]. However, most cyanobacterial sequences obtained from these intervals could not be affiliated to a given genus, and those that were affiliated mainly belong to Cyanobium, which seems to lack N-fixing genes [69]. Some work on fossil sedimentary DNA possibly dovetailed with characteristic pigment analysis could therefore reveal information on the evolution of Lake Ohrid’s productivity and planktonic communities in relation with Quaternary changes of nutrient availability.

High χARM/SIRM is found in the top samples at 1.8 and 4.7 m. Those two share similar OTUs that allowed them to integrate into a similarity group at the 97% OTU cutoff (Figure 6C). Just et al., 2020 hypothesized that these high values could be related to the production of bacterial magnetite and greigite, given the absence of observed changes in lithogenic magnetic minerals in this section. Our data do not highlight the presence of magnetotactic bacteria. However, these samples form one of the rare groups that show a contribution by methanogenic Archaea of the Euryarchaeota phylum (Figure 6B,C), and a low but significant contribution by sulfate reducers (Figure 6C), which could support an active S cycle or organic matter transformation involving magnetite or greigite formation. This is also the case, to a lesser extent, for the group formed by samples at 12.4 and 29.1 m, which are marked by high sedimentary S concentrations (Figure 2). Sulfur is interpreted as a significant environmental parameter influencing the observed subsurface diversity of Lake Ohrid (Figure 9A,D,G). Being fed primarily by karst springs draining marine Mesozoic limestones, Lake Ohrid receives variable but constant supply of sulfate on glacial–interglacial time-scales. Similar to the supply of Ca and CO_3_ ions, it is expected that the supply of SO_4_ is reduced during glacials when soil formation, weathering and karst activity is reduced [40]. However, the ubiquity of SO_4_ in Lake Ohrid is likely to support an active S cycle, even during the last glacial period, as observed in particular between 12.4 m and 29.1 m (Figure 8).

### 5.4. Lake Ohrid Specificity

Lake Ohrid is characterized by marked changes in sedimentary composition between glacial and interglacial periods [2,5], which contribute to the use of the Lake Ohrid sedimentary record for powerful paleoclimatic reconstructions (e.g., [1]). However, the study of the lake biosphere also highlights the strong resilience of the planktonic to benthic communities to major climatic events [14,15]. Based on the few environmental data available, it is not possible to disentangle the parameters controlling microbial diversity in the sediments. While bulk sedimentary XRF and magnetic data can provide key information regarding sedimentary processes at a macroscale, they lack the second order precision that could help unravel early diagenetic processes. The latter could be better addressed using, for example, pore water chemistry and its stable isotope composition, which were not available for our study. 

Links with changes in diagenetic conditions, identified by Just et al. (2016), could not be confirmed. Based on a difference in early diagenetic precipitates (shifts from ferrimagnetic iron sulfides to ferro-carbonate siderites at 320 ka, ca. 140 m), the authors suggested higher sulfate concentration in the lake before 320 ka. This would have permitted a deeper penetration of sulfate in the sediment and favored formation of iron sulfide via sulfate reduction. After 320 ka, rapid depletion of sulfate in the shallow sediments of the lake may have permitted the formation of siderite (instead of Fe-sulfide) through methanogenesis dominance in the shallow sediments. We observed a general increase of potential sulfate reducers between 30.83 ka and 316.43 ka (although samples at 39.9, 68.8 83.5 and 109.5 m do not bear this signal). Before 320 ka, no peak in potential sulfate reducers nor methanogens could be identified. While the METAGENassist results must be taken with caution for such inferences, no obvious shift between methane-related vs. sulfur-related clades were observed, based on varied OTUs cutoffs. This can be due to a suppression of the potential methanogenic or sulfate reducer genetic signatures with time. The sulfate-methane transition zone is indeed generally constrained to the first centimeter of the sediment [32,33] and while some signatures could be retained with burial [25], the continued microbial activity in the deep sediment may lead to turnover of the dominant communities and overall suppression of the initial signal. We may also miss their presence through the use of non-specific 16S rRNA gene sequencing. Targeting and quantifying functional genes associated with sulfate reduction (dsrA) or methanogeneis (mcrA) in the archived DNA pool of the deep Ohrid sediment could provide valuable insights on this question but could not be successfully applied in our system given the limited quality and quantity of obtained DNA.

Interestingly, samples older than 320 ka indeed support different metabolic potential than younger ones. In particular, hydrogen production and carbon dioxide fixation are the main metabolisms highlighted by our simulation (Figure 8). The extent to which this might be related to a change in cycling from sulfate- to methane-driven microbial cycling in the first place remains unresolved. The lack of labile OM available as a carbon source for deep sedimentary communities below 135 m may lead to a shift towards more dominant chemolithoautotrophic metabolisms. The conjunction of H_2_ production along with CO_2_ fixation points towards a potential niche for hydrogenotrophic methanogenesis or acetogenesis. Such processes have been suggested in the past for deep lacustrine sediments [70], deep marine sediments and hydrothermal systems [71,72].

Intracellular vs. extracellular DNA extraction methods have shown their value in the study of deep life in lacustrine settings [61]. These methods would be necessary to confirm that the Cyanobacteria and Gammaproteobacteria-related OTUs significantly influencing the compositions of samples from 9.6 m and 147.9 m are inherited from dead cell biomass. It would also allow discrimination between transported-archived vs. active-dormant living microbes in the deep sediment of Lake Ohrid, since spore-forming or motility abilities seem to increase with depth. This is in line with a substantial impact of erosional processes also observable for various samples originating from dry and cold (glacial) periods, on the Ohrid subsurface biosphere.

Our study of the influence of environmental parameters on the subsurface biosphere composition on Lake Ohrid highlights a strong impact of erosion and transport on the sedimentary DNA pool. Nevertheless, a significant part of the community diversity is held by phylotypes adapted to low energy environments, which suggests that the Lake Ohrid deep biosphere would be adapted to survive [73] until ca. 515 ka (ca. 200 mblf), and that these phylotypes have partly erased a potential microbial signature that could have been inherited through paleoclimatic conditions. 

## 6. Conclusions

Based on 16S rRNA gene sequences, the subsurface biosphere composition of Lake Ohrid is dominated by energy conservative microbial communities common to deep sedimentary settings, regardless of their marine or lacustrine origin. Bathyarchaeia and Atribacteria play a strong role in structuring this subsurface community beta-diversity. The ability of these communities to adapt to low energy environments has likely erased the potential original paleoenvironmental, paleolimnological and early diagenetic signals that Lake Ohrid sediments have recorded, except for water column or soil DNA archiving during dry glacial periods. Unlike other lacustrine systems, it seems that the strong resilience of Lake Ohrid’s ecosystem and/or the peculiar limnological characteristics of this lake basin do not allow for the conservation or transfer of a specific microbial community in these sedimentary archives. 

## Supplementary Materials

The following are available online at https://www.mdpi.com/2076-2607/8/11/1736/s1, Table S1: Climatic proxy matrix, Table S2: Elemental composition matrix, Table S3: Magnetic parameters matrix, Table S4: OTU level population matrix, Table S5: Phylum level population matrix.
